# Supporting appropriate use of extended dual antiplatelet therapy post-myocardial infarction based on an innovative 12-month ticagrelor virtual service

**DOI:** 10.3389/fcvm.2024.1399899

**Published:** 2024-09-05

**Authors:** Rani Khatib, Abigail Barrowcliff, Franki Wilson, Sidra Awan, Mutiba Khan, Stephen Wheatcroft, Alistair S. Hall

**Affiliations:** ^1^ Cardiology Department, Leeds Teaching Hospitals NHS Trust, Leeds, United Kingdom; ^2^ Leeds Institute of Cardiovascular and Metabolic Medicine, University of Leeds, Leeds, United Kingdom; ^3^ Medicines Management, Leeds Teaching Hospitals NHS Trust, Leeds, United Kingdom

**Keywords:** dual antiplatelet therapy, myocardial infarction, P2Y12 receptor antagonist, ticagrelor, guidelines, medicines optimisation, bleeding risk

## Abstract

**Purpose:**

Extended dual antiplatelet therapy (DAPT) with ticagrelor and aspirin is recommended in selected cases after myocardial infarction (MI) but not widely deployed in practice. This study assessed an innovative, cardiology pharmacist-led virtual service for determining eligibility for extended DAPT among patients completing 12 months of initial DAPT in primary care following MI.

**Methods:**

Within this model, potentially eligible individuals are reviewed virtually by a cardiology pharmacist for suitability for extended DAPT with reduced-dose ticagrelor [60 mg twice daily (BD)] for up to 3 years. Eligibility is guided by the PEGASUS-TIMI 54 trial criteria (aged ≥50 years and having ≥1 high-risk feature for further ischaemic events). This is balanced against potential ineligibility driven primarily by bleeding risk, assessed using PRECISE-DAPT score. The final recommendation is sent to primary care to action. The present work is a retrospective evaluation of patients referred to the service between July 2018 and December 2021.

**Results:**

A total of 200 patients were included [*n* = 131 (65.5%) male; mean age: 69.4 ± 9.5 years]. Of these, 79 (39.5%) were recommended for extended DAPT based on the balance of risks for further ischaemic events vs. bleeding. Sixty-three patients on high-dose DAPT (ticagrelor 90 mg BD)—which is inappropriate beyond 12 months—were reassigned to reduced-dose DAPT or aspirin monotherapy.

**Conclusions:**

This virtual clinic played a key role in medicines optimisation, enabling appropriate patients to benefit from extended DAPT while offsetting bleeding risk. The model could be adapted locally for use elsewhere.

## 1 Introduction

Ticagrelor is a P2Y12 receptor antagonist commonly used to inhibit platelet aggregation and prevent atherothrombosis. It has been approved for co-administration with aspirin in patients with acute coronary syndrome or with a history of myocardial infarction (MI) and a high risk of developing an atherothrombotic event (1).

Extended dual antiplatelet therapy (DAPT) with ticagrelor plus aspirin—beyond 12 months after the index MI—is an important option in selected patients. In the PEGASUS-TIMI 54 study of more than 20,000 subjects aged ≥50 years who had experienced an MI more than 12 months previously, extended DAPT with ticagrelor 60 mg twice daily (BD) for 3 years plus low-dose aspirin significantly reduced the risk of cardiovascular death, MI or stroke, compared with aspirin alone [hazard ratio: 0.84; 95% confidence interval (CI): 0.74–0.95; *P* = 0.004] (2). However, rates of major bleeding were higher with ticagrelor 60 mg than with placebo (2.30% vs. 1.06%, respectively; *P* < 0.001) (2). Thus, there is a need for practitioners to balance the benefits of extended DAPT with the associated risks.

The European Society of Cardiology (ESC) and the UK National Institute for Health and Care Excellence (NICE) have published guidance on extended DAPT with ticagrelor plus aspirin (3, 4). NICE only recommends ticagrelor (as reflected in the licensing) but the ESC also recommends other antiplatelets, which can be used instead for extended DAPT. In October 2023, the ESC published new acute coronary syndromes (ACS) management guidelines, which put further emphasis on long-term antithrombotic treatment strategies, and also provide criteria for assessing thrombotic risk (5). However, these were not available at the time of the present study.

In UK routine practice, a recommendation on extended DAPT beyond 12 months is not usually made when first initiating DAPT after the index MI. Patients will receive a follow up review one month post MI and the majority discharged prior to reaching 12 months post MI. This can be problematic because most patients are managed in primary care—and are no longer under active follow up by the cardiology multidisciplinary team (MDT)—at the time a judgement on extended DAPT needs to be made. Such decision making can be challenging because it requires both an assessment of the risk of a further ischaemic event (based on the PEGASUS-TIMI 54 trial criteria), as well as evaluation of the countervailing risk of bleeding events. As a result, the current standard of care in the UK does not lend itself to offering treatment with extended DAPT and the data reflects this with a low uptake in ticagrelor 60 mg as can be seen in the Supplementary Material.

Current NICE guidance does not specify how a bleeding risk assessment should be undertaken (4), and there is no recognised, standardised tool for this. In our practice, we use the five-point PRECISE-DAPT risk calculator. PRECISE-DAPT was originally designed to provide a standardised method for predicting bleeding risk in patients undergoing stent implantation and subsequent DAPT (6, 7), and it is an ESC-recommended tool in this setting (3). However, not all patients undergo a bleeding risk assessment at the index event and, in any case, follow-up scores appear to be a better predictor of risk than baseline calculations (8). This suggests that PRECISE-DAPT could be a reliable tool even when used at 12 months. It is important to consider the individual patient's complete clinical picture during bleeding risk assessment.

To help optimise decision making around extended DAPT with ticagrelor—and facilitate the implementation of current guidelines—we have introduced an innovative virtual clinic at 12-months post-discharge. This service aims to review patients within primary care in the Leeds area who are on ticagrelor-based DAPT and are due to complete (or have recently completed) their initial 12 months of treatment. The present paper describes the model employed and provides data on an initial group of patients.

## 2 Materials and methods

### 2.1 Service background and design

**Figure 1:**
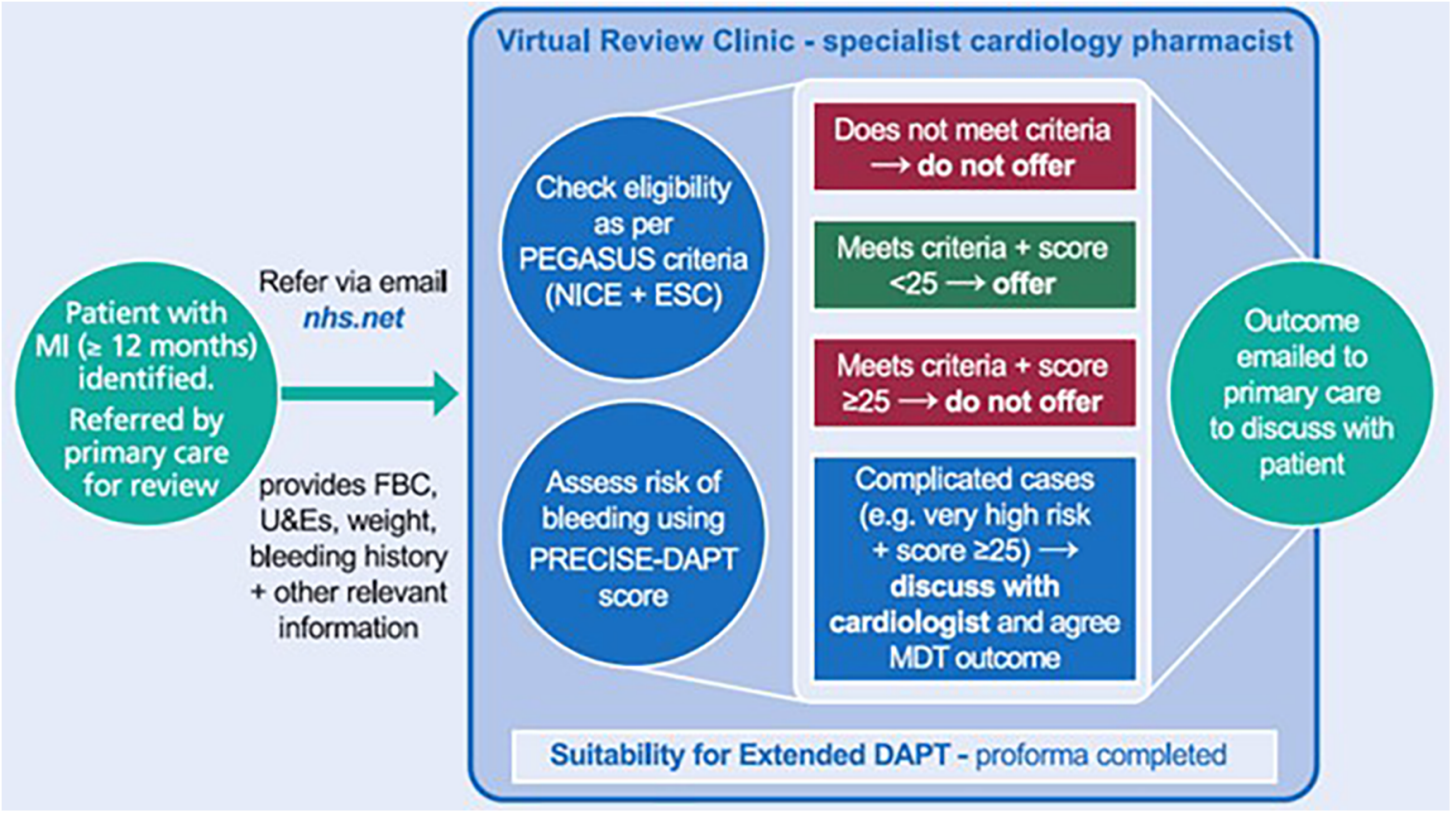
Extended DAPT virtual review clinic. DAPT, dual antiplatelet therapy; ESC, European Society of Cardiology; MDT, multidisciplinary team; FBC, full blood count; MI, myocardial infarction; NICE, National Institute for Health and Care Excellence; U&E, urea and electrolytes.

The NHS Leeds Clinical Commissioning Groups Partnership commissioned a consultant cardiology pharmacist from the Leeds Teaching Hospitals NHS Trust to support the review of patients who were potentially eligible for extended DAPT. Working alongside colleagues from primary care, an MDT of cardiologists and cardiology pharmacists developed guidance and a new clinical pathway (Figure 1) (9). Specifically, a novel virtual review clinic was established. Guidance was then disseminated to primary care practitioners, including physicians, nurses and pharmacists. These guidelines were designed to support primary care practitioners in making prescribing decisions based on the principles of a shared decision-making approach with patients. They were encouraged to refer any candidates they were not confident to manage in the community.

The pathway is based on a three-step process. First, primary care teams identify potentially eligible patients as part of the standard 12-month post-MI review process; any individuals that they feel unable to manage themselves can be referred to the service. This is done via secure e-mail from the primary care team to the virtual clinic. The referring practitioner must provide all of the necessary information, including full blood counts, urea and electrolyte measurements, weight, bleeding history, and other details relevant to decision making on extended DAPT. The NHS e-mail network is used for this process, which is a secure system to ensure patient confidentiality.

Second, patients are reviewed virtually by a cardiology pharmacist against the agreed protocol for identifying individuals suitable for extended DAPT with ticagrelor 60 mg BD for up to 3 years [as per the current license (1) and recommendations from NICE (4) and the ESC (3)], alongside lifelong aspirin 75 mg once daily (OD). Their potential eligibility is guided by whether the individual meets the PEGASUS-TIMI 54 criteria of being aged ≥50 years and having one or more high-risk feature for further ischaemic events (2): age ≥65 years; diabetes mellitus requiring medication; a second prior spontaneous MI; multivessel coronary artery disease (defined as two or more vessels with ≥50% stenosis); or non-end-stage chronic kidney disease [CKD; estimated creatinine clearance (CrCl) <60 ml/min]. Criteria for ineligibility for extended DAPT are derived from the exclusion criteria from the PEGASUS-TIMI 54 study: planned use of a P2Y12 antagonist other than ticagrelor (e.g., clopidogrel, prasugrel), dipyridamole, cilostazol, or anticoagulation; history of gastrointestinal bleeding in the past 6 months or a bleeding disorder; major surgery in the past 30 days; central nervous system tumour, intracranial vascular abnormality, or prior intracranial haemorrhage; clinically significant bradycardia during the 12 months while on ticagrelor and dialysis or severe liver disease; or end-stage CKD. Based on discussions with nephrologists, it was decided locally that patients active on the kidney transplant waiting list (for potential pre-emptive kidney transplantation) should also be ineligible. Patients treated with a coronary artery bypass graft (CABG) intervention within the past 5 years were excluded from PEGASUS, unless they had an MI subsequent to CABG. The ESC recommends considering extended DAPT 12 months post-CABG, but such use is off-license.

Patients are also assessed for their risk of bleeding using the PRECISE-DAPT method, which provides a risk score based on five criteria: age, CrCl, haemoglobin levels, white blood cell count, and prior spontaneous bleeding (6, 7). We also look at any bleeding history, intolerance or non-adherence issues in the past 12 months during initial DAPT treatment (with ticagrelor 90 mg BD or other). Blood test results are required to be recent (≤1 month old); results from the index admission are not used. A PRECISE-DAPT score of ≥25 indicates a high risk of bleeding. In the risk score derivation cohort, a patient with a PRECISE-DAPT score of 25 had a TIMI major or minor bleeding risk of 1.8% and TIMI major bleeding risk of 1.0% within 1 year (6). This increased to 2.7% and 1.4%, respectively, when PRECISE-DAPT score increased to 30 (6).

Patients who meet the PEGASUS-TIMI 54 criteria and have a PRECISE-DAPT score <25 are considered to be potentially eligible for extended DAPT. Individuals who do not meet the PEGASUS-TIMI 54 criteria and/or have a PRECISE-DAPT score ≥25 are typically considered to be ineligible; however, some clinical judgement can be exercised in complicated cases, for example in patients with a very high risk of further ischaemic events who also have a PRECISE-DAPT score ≥25. In these instances, the case may be discussed with a cardiologist and a joint MDT recommendation is made; treatment options and their respective risks/benefits are then discussed with the patient to allow shared decision making, and any final advice to initiate is accompanied by guidance on regular monitoring. If extended DAPT is not recommended, patients are advised to continue aspirin 75 mg OD monotherapy lifelong (provided there are no contraindications).

Clopidogrel can be considered for extended DAPT if the patient is unable to tolerate ticagrelor. More recently, prasugrel and the use of dual pathway inhibition (rivaroxaban 2.5 mg BD) have also entered the clinical pathway, alongside aspirin 75 mg.

A proforma was developed to standardise information collection and assessment. It includes patient information (e.g., index MI details, dates, angiogram report, any recommendations from the interventionalist, etc.), eligibility criteria, PRECISE-DAPT score, and subsequent recommendations on extended DAPT.

Finally, an e-mail (with the proforma attached) outlining the final recommendation is sent back to the primary care practitioner who can then offer that recommendation to the patient. The proforma is also uploaded to the Electronic Patient Record (PPM+), an electronic system used in Leeds to record healthcare information. This system allows primary and secondary care practitioners to access inpatient and outpatient clinical notes.

### 2.2 Assessments

The present work is a retrospective evaluation of patients referred to the service between July 2018 and December 2021. The analysis was conducted in accordance with the Declaration of Helsinki. As this was a service development programme, Ethics Committee approval was not required, in line with local policy. Patient consent was not needed because only anonymised data was reported.

Data were gathered using the “Suitability for Extended DAPT” proforma (see Supplementary Material) and the electronic medical records database. All reviews were analysed by one individual (for consistency) and were checked by a consultant cardiology pharmacist.

The data reported here include baseline characteristics, PEGASUS-TIMI 54 high-risk criteria, PRECISE-DAPT scores, and antiplatelet regimens at the time of referral and following virtual review.

### 2.3 Consent

No patient identifiable data are included.

### 2.4 Statistical analyses

Descriptive statistics are provided, including mean, standard deviation and range for continuous variables, and frequency and percentage for categorical variables.

## 3 Results

A total of 200 patients were reviewed in the virtual clinic, of whom 131 (65.5%) were male and 69 (34.5%) were female (Table 1). The mean age was 69.4 ± 9.5 years (range: 37–91 years). One hundred and twenty-two (61.0%) had experienced a non-ST segment elevation MI, and 69 (34.5%) had had an ST segment elevation MI.

**Table 1:** Baseline characteristics.

Characteristic	Patients (*N* = 200)
Sex, *n* (%)	
Male	131 (65.5)
Female	69 (34.5)
Age, years, mean (SD, range)	69.4 (9.5; 37–91)
Disease type, *n* (%)	
NSTEMI	122 (61.0)
STEMI	69 (34.5)
Unstable angina	3 (1.5)
Other ACS	6 (3.0)

ACS, acute coronary syndrome; MI, myocardial infarction; NSTEMI, non-ST segment elevation MI; SD, standard deviation; STEMI, ST segment elevation MI.

With regard to the PEGASUS-TIMI 54 criteria for high risk of further ischaemic events, 183 patients (91.5%) had at least one risk factor, the most common of which were age ≥65 years (*n* = 128; 64.0%) and multivessel coronary artery disease (*n* = 120; 60.0%) (Table 2). Of the remaining patients, 15 (7.5%) were considered to be ineligible and not offered extended DAPT. Two patients (1.0%) were not eligible according to PEGASUS-TIMI 54 criteria, but were offered it after discussion with an interventionist.

**Table 2:** Risk factors for further ischaemic events (as per PEGASUS-TIMI 54).

Risk factors	Patients (*N* = 200)
One or more risk factors	183 (91.5)
Age ≥65 years	128 (64.0)
Diabetes mellitus requiring medication	51 (25.5)
Second prior MI	43 (21.5)
Multivessel coronary artery disease	120 (60.0)
CrCl <60 ml/min	51 (25.5)

Data are *n* (%). Some patients had more than one risk factor. CrCl, estimated creatinine clearance; MI, myocardial infarction.

Calculation of PRECISE-DAPT scores demonstrated that 134 patients (67.0% of all patients) were not at high risk of bleeding (score <25) and were therefore potentially eligible for extended DAPT. Around half (*n* = 66/134; 49.3%) were ultimately offered this treatment based on assessment of PEGASUS-TIMI 54 criteria (Table 3). The other 68 individuals were not offered extended DAPT for various reasons, most commonly because they had received a coronary artery bypass graft following the index MI (and were therefore ineligible as per the PEGASUS-TIMI 54 exclusion criteria).

**Table 3:** PRECISE-DAPT scores.

PRECISE-DAPT score	Offered DAPT at 12-month review		Not offered DAPT at 12-month review	
	*n*, %	Mean number of risk factors^a^	*n*, %	Mean number of risk factors^a^
≥25 (*N* = 64^b^)	13 (20.3)	3.5	50 (78.1)	2.5
<25 (*N* = 134)	66 (49.3)	1.8	68 (50.7)	1.3

*N* = 198 (blood test results were not available for 2 patients, neither of whom was offered extended DAPT). ^a^As per the PEGASUS-TIMI 54 risk criteria. ^b^One patient with PRECISE-DAPT score ≥25 was referred too early (before completing 12 months post-index MI) and hence no recommendation was made. DAPT, dual antiplatelet therapy; MI, myocardial infarction.

Blood test results were not available for 2 patients, and neither was offered extended DAPT. The remaining 64 patients (32.0%) had a high risk of bleeding (PRECISE-DAPT score ≥25) and most of these (*n* = 50/64; 78.1%) were therefore considered to be ineligible for extended DAPT; however, a small number of individuals (*n* = 13/64; 20.3%) were recommended for extended DAPT following discussion with a cardiologist, due to a particularly high risk of further ischaemic events.

**Figure 2:**
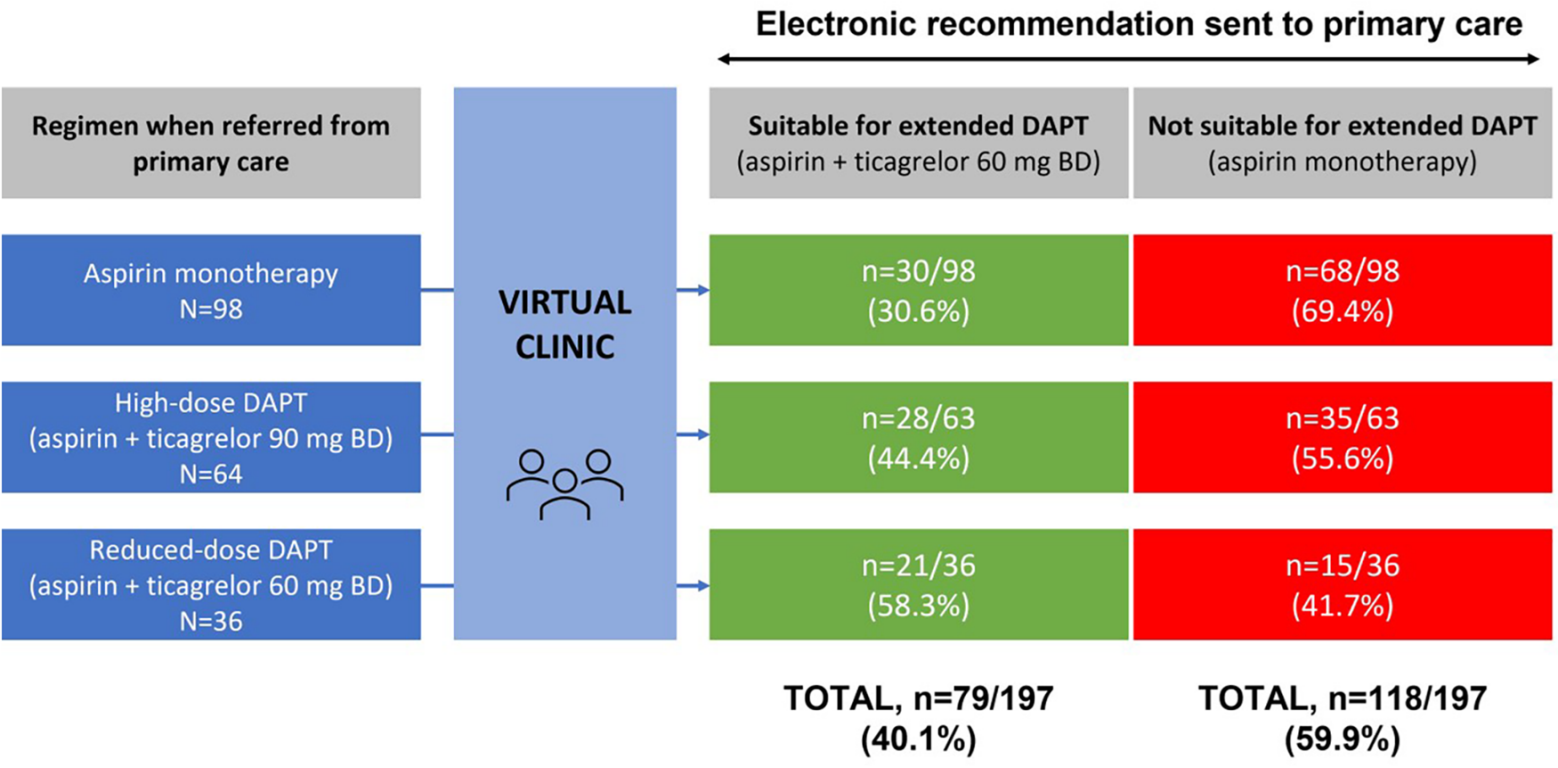
Antiplatelet regimens before and after virtual review clinic. Data were analysed from 200 patients. In 2 cases, it was unclear which regimen they were on when referred from primary care; in addition, 1 patient on high-dose DAPT was referred to the service too early (before completing 12 months post-index MI) and hence no recommendation was made either way. BD, twice daily; DAPT, dual antiplatelet therapy; MI, myocardial infarction.

Overall, 79 patients (39.5%) were recommended for extended DAPT based on the virtual clinic review. Antiplatelet regimens before and after this review are shown in Figure 2. Ninety-eight individuals had been on aspirin monotherapy prior to virtual review, and of these, 30 (30.6%) were recommended for extended DAPT. A further 64 patients had previously been on high-dose ticagrelor (90 mg BD) plus aspirin; this was discontinued in all but one case (as the patient was yet to complete 12 months post-index MI), with 28 (44.4%) being recommended for extended DAPT with reduced-dose ticagrelor (60 mg BD). In total, 36 patients were already on DAPT with reduced-dose ticagrelor plus aspirin prior to virtual review, and continuation of this regimen was recommended for 21 individuals (58.3%).

Among the 64 patients referred on ticagrelor 90 mg BD, the mean duration of treatment beyond the licensed 12 months was 5 ± 6.7 months; 10 patients had been on this regimen inappropriately for more than 1 year, and 1 individual had been on it for nearly 2 years longer than recommended and had a PRECISE-DAPT score of 34.

Most of the 200 patients included in this analysis had received no recommendation from the interventionalist when discharged post-index event on whether or not DAPT should be extended beyond 12 months (*n* = 159; 79.5%). The remaining 41 (20.5%) did have a recommendation either way, but this was reversed at the virtual review clinic in 7 cases. In 4 of these instances, a recommendation to treat with extended DAPT was overturned (2 did not meet PEGASUS-TIMI 54 criteria, 1 suffered shortness of breath with ticagrelor, and 1 had ongoing anaemia and general poor health). In the other 3 cases, extended DAPT was specifically not recommended, but we decided to offer it to 1 patient as an exception due to stent thrombosis; 1 who was not previously recommended due to bruising, but who had high risk factors; and 1 who was offered due to several risk factors.

## 4 Discussion

An innovative virtual clinic has been developed for improving decision making around extended DAPT with ticagrelor at 12 months post-discharge for MI. Potentially eligible patients are identified in primary care and a medication review performed virtually by an advanced cardiology pharmacist in secondary care against predefined criteria for identifying those that are suitable for extended DAPT with ticagrelor 60 mg BD for 3 years plus lifelong aspirin 75 mg OD.

In an initial group of 200 patients reviewed within this clinic, most (79.5%) had no recommendation from when they were first discharged on whether or not they should receive extended DAPT. Primary care practitioners were encouraged to refer individuals they were not confident to manage themselves, and this may have enriched the cohort with patients lacking a clear discharge recommendation. Nonetheless, the virtual review clinic played an essential role in ensuring that appropriate decision making was undertaken with these individuals; without it, it is likely that some patients would not have been offered extended DAPT in accordance with national and international guidelines (3, 4). Furthermore, among the 41 individuals with a recommendation on extended DAPT at discharge (either for or against), that recommendation was reversed in 7 cases. This is not surprising given that eligibility and bleeding risk characteristics can change significantly for any given patient over the course of a year, and hence the original recommendation will not always remain suitable. Thus, a 12-month review is important for ensuring that the right patients receive extended DAPT. Importantly, we also provide local guidelines on assessing bleeding risk, and these are available to primary care teams, thus facilitating subsequent de-escalation of treatment should bleeding risk increase once patients have left our service.

It is also notable that the 12-month virtual clinic recommended a treatment change in the majority of cases: 30 patients were “upgraded” from aspirin monotherapy to extended DAPT; 15 patients were “downgraded” from reduced-dose DAPT (ticagrelor 60 mg BD + aspirin) to aspirin monotherapy; and all 63 individuals on high-dose DAPT (ticagrelor 90 mg BD + aspirin) for over 12 months were reassigned to reduced-dose DAPT or aspirin monotherapy (Figure 2). Ensuring that patients do not continue high-dose ticagrelor beyond 12 months is a particularly important benefit of the clinic given the associated risk of bleeding. A recent meta-analysis found that although extending DAPT beyond 12 months enhances thrombotic protection, there is no effect on mortality due to the increased risk of major bleeding complications (10). Information is currently lacking on how many patients are inadvertently continued on high-dose DAPT beyond 12 months, with no assessment of bleeding risk and/or switch to ticagrelor 60 mg BD. It is possible that there is less caution around extending DAPT beyond 12 months since the publication of PEGASUS-TIMI 54 and subsequent changes in NICE guidance.

A key aspect of our service is the appraisal of bleeding risk, to ensure appropriate use of extended DAPT. Current guidance from NICE does not specify how this should be performed (4). In our virtual clinic model, we employ the simple-to-use, standardised, five-point PRECISE-DAPT risk score. This tool was originally designed for use at the time of the index stent procedure (6, 7), and it is recommended by the ESC in such settings (3). The PRECISE-DAPT tool has been used in studies of prolonged DAPT (≥12 months), and a longer duration of DAPT was found to significantly increase the bleeding risk only in patients with a score ≥25 (11). PRECISE-DAPT has also been shown to have good predictive value for long-term bleeding events (up to 5 years) (12). Furthermore, in a recent analysis of 480 patients on DAPT, PRECISE-DAPT score was not constant and indeed changed significantly over time in many cases; in a multivariate analysis, follow-up PRECISE-DAPT scores rather than baseline assessments were found to be an independent predictor of bleeding (8). This suggests that PRECISE-DAPT may be a reliable tool for assessing bleeding risk even when evaluated at 12 months post-index event.

From a practical perspective, we believe that our clinic model is innovative and has important advantages in: (i) supporting treatment decision making among primary care practitioners (who may not always be best qualified or confident to make an assessment on eligibility for extended DAPT); (ii) maintaining a low burden on patients (given that the clinic is virtual); and (iii) minimising the impact on cardiology outpatient services due to its virtual nature, and on cardiologists because the clinic is led primarily by cardiology pharmacists. Cardiologists can also refer primary care “Advice and Guidance” requests around extended DAPT to this clinic, saving them further time.

Importantly, the cardiology pharmacists running these clinics have advanced knowledge, training and experience, and also have access to the full suite of clinical information required for detailed assessment. Nonetheless, it is important to note that support from cardiologists is available whenever necessary—particularly around cases for which decision making is complex, such as patients with a high risk of further ischaemic events who also have a high bleeding risk. Furthermore, all other elements of normal post-MI care remain in place; our service is additional to and not a replacement for these.

To the best of our knowledge, this is the first study to assess an innovative pharmacist-led virtual model of decision making around extended DAPT with ticagrelor. However, it is underpinned by established practice methods. At our centre, we have been running pharmacist-led medicines optimisation clinics for post-MI patients for many years, with proven benefits in optimising secondary prevention medicine prescribing, increasing adherence rates, and decreasing hospital readmissions (13). We also carry out proprotein convertase subtilisin/kexin type 9 inhibitor (PCSK9i) clinics led by pharmacists, and these have delivered significant lipid-lowering benefits in eligible patients (14). Meanwhile, the use of virtual review methods is increasing within the field of cardiology (15, 16), and this trend is likely to continue in the future.

Importantly, our model is sufficiently flexible to integrate changes in best practice. Since the present data were collected, other treatments have demonstrated clinical benefits in advanced trials. In particular, prasugrel has proven efficacious for the initial 12 months of DAPT post-MI in combination with aspirin (17) [and is now recommended in ESC guidelines instead of ticagrelor in this setting (5)], and rivaroxaban has been studied as extended therapy in eligible patients after the initial 12 months of DAPT (18). These have now been incorporated into our virtual clinic review process. Indeed, the availability of rivaroxaban 2.5 mg BD provides an alternative option in some high-risk patients who may be ineligible for ticagrelor (e.g., those treated with CABG at the index event or who are unable to tolerate ticagrelor). Bleeding risk is assessed in the same way in such individuals. The availability of these additional options may further complicate decision making, highlighting the key role of our service in supporting primary care practitioners.

Furthermore, following on from the success of the model based on referral from primary care, we have now expanded the scope of the clinic with the aim of proactively identifying eligible patients within the medicines optimisation team in secondary care; since early 2022, we have been reviewing at 11 months post-index MI those patients who were referred to us shortly after their index event. We work closely with post-MI clinics (both cardiology pharmacist- and cardiologist-led), who refer eligible patients after their appointment at 4–8 weeks post-index event, to add to our future clinic list for review. With these individuals, the aim is to prevent unnecessary continuation of ticagrelor 90 mg BD and ensure the continuation of an appropriate treatment plan developed at the start of post-MI management, rather than performing a reactive intervention. This is particularly important given the number of patients continuing on ticagrelor 90 mg BD inappropriately beyond 12 months. Sixty-four such patients in the current study had been on this dose for a mean of 5 months too long, and 10 individuals had been on it for >12 months too long. A longer duration of DAPT (>12 months) is associated with a significantly increased risk of TIMI major bleeding (19).

We must acknowledge the limitations of the present work. First, referral into the service was at the discretion of primary care teams. It was based on an “opt-in” model and a number of variables could have affected whether or not patients were referred. It is unclear how many had a decision made without referral, and how many were not referred when they could have been. This issue has now been resolved with prospective identification of eligible patients. Second, the PRECISE-DAPT tool has not been validated for use at 12 months post-event, and most likely overestimates bleeding risk in these individuals because they have all tolerated 12 months of DAPT prior to assessment. Most bleeding events occur within the first few months of DAPT (20), and the present group represents a selected population who may be at lower risk of bleeding with extended DAPT. Nonetheless, as already described, we believe PRECISE-DAPT to be an effective tool in this setting and the model allows for a degree of clinical judgement in confirming eligibility for extended DAPT. Third, a lack of direct patient contact could be considered a limitation of the model; a conversation is still required to discuss risks and benefits, and to facilitate informed decision making in consultation with the patient. Fourth, no long-term outcome data were collected and it would be valuable in future to assess the impact of the clinic on subsequent rates of ischaemic events and bleeding. However, the focus of this paper was on medicines optimisation and better implementation of guidelines rather than outcomes, and in this regard, it is notable that nationwide prescribing data suggest our region is the second highest user of extended DAPT (by crude volume) across England (21). Please see Supplementary Material. Finally, it would also be useful to collect data from GP surgeries to see how the service has benefitted them. Nonetheless, we have completed an important first step in demonstrating that the model supports medicines optimisation with ticagrelor.

In conclusion, we have developed an innovative virtual clinic at 12-months post-discharge, designed to help optimise decision making around extended DAPT with ticagrelor and facilitate the implementation of current guidelines. In an initial cohort of 200 patients, the clinic played an important role in medicines optimisation, enabling more patients to benefit from extended DAPT while also balancing the risk of bleeding events. Our service offers a model that could be locally adapted for use elsewhere in the UK and beyond.

## Data Availability

The raw data supporting the conclusions of this article will be made available by the authors, without undue reservation.
