# Supplementary material for: Supporting appropriate use of extended dual antiplatelet therapy post-myocardial infarction based on an innovative 12-month ticagrelor virtual service
Source: Front Cardiovasc Med. 2024 Sep 5;11:1399899. doi: 10.3389/fcvm.2024.1399899 (PMC11417623; doi:10.3389/fcvm.2024.1399899)

## Supplementary Material

The data extracted and presented in the bar chart below highlights the differences across England in the uptake of ticagrelor 60mg by crude volume(1).

The uptake of extended DAPT is broadly quite low in England and our locality (West Yorkshire Integrated Care Board (ICB)) is outstanding in comparison. We are the second highest user across the country. This is potentially due to the extended DAPT service we provide.

Sheffield and Rotherham, from the South Yorkshire ICB, were two of the sites for the PEGASUS study.

## References


(1) Open Prescribing. Search GP prescribing data. https://openprescribing.net/analyse/ [Accessed August 5, 2024].

(2) Clinicaltrials.gov. Prevention of Cardiovascular Events (eg, Death From Heart or Vascular Disease, Heart Attack, or Stroke) in Patients With Prior Heart Attack Using Ticagrelor Compared to Placebo on a Background of Aspirin (PEGASUS). https://clinicaltrials.gov/study/NCT01225562/ [Accessed August 7, 2024]


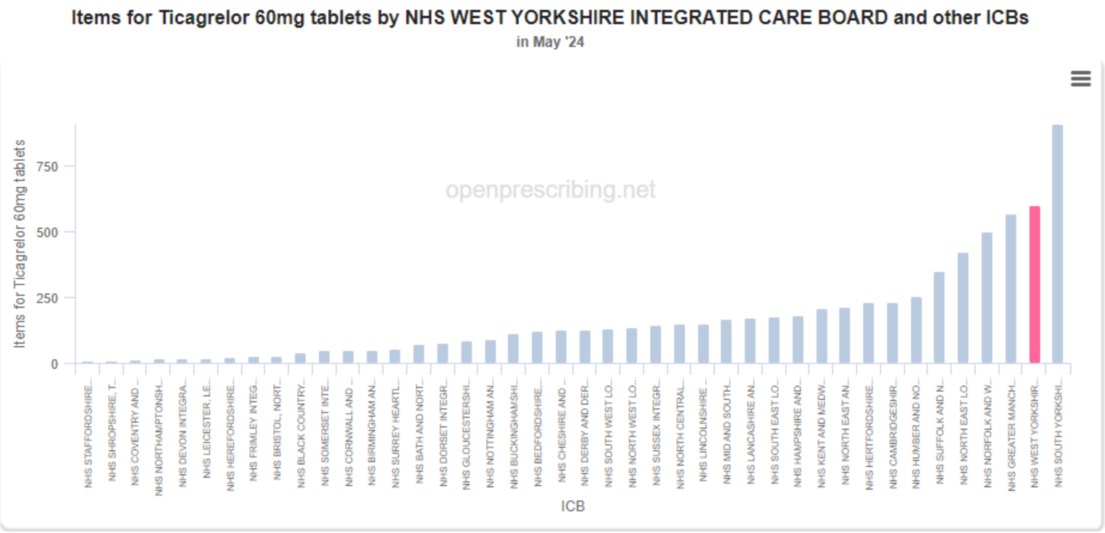

Supplement: Supplementary file 1 [file Datasheet1.pdf]
