# Supplementary material for: Supporting appropriate use of extended dual antiplatelet therapy post-myocardial infarction based on an innovative 12-month ticagrelor virtual service
Source: Front Cardiovasc Med. 2024 Sep 5;11:1399899. doi: 10.3389/fcvm.2024.1399899 (PMC11417623; doi:10.3389/fcvm.2024.1399899)
Supplement: Supplementary file 2 [file Datasheet2.pdf]

# Suitability for Extended DAPT

**Patient: NHS number: D.O.B: Weight:**

**Indication:**

- Diagnosis, date, any intervention
- Any past cardiac Hx
- Angio:
- Dates on ticagrelor, any issues
- Recommendation made by interventionist regarding extended DAPT

**Other**

- EF, anaemia, bleeding, drug interactions

## Risk Factors:

| Assessment of patient against criteria | Criteria |
|---|---|
| | age >=65 years |
| | diabetes mellitus requiring medication |
| | a second prior spontaneous myocardial infarction |
| | multi-vessel coronary artery disease |
| | estimated CrCl < 60 mL/min |

**Exclusion:**
See assumptions.

## Bleeding Risk:

**Based on FBC & U&Es (date)**

**<u>Recommendations:</u>**
Weigh up risk factors and bleeding risk.

**<u>Next</u>**
**Extended DAPT can be offered (with ticagrelor 60mg for 3 years)/ is not recommended at this time. Any additional monitoring e.g. FBC.**

If extended DAPT is recommended then GP should ensure that assumptions are correct.
Discuss with patient the recommendations made, based on the principles of shared decision-making approach.

## Assumptions (ensure these are correct):

Patient has no bleeding disorder or melena
There is no History of central nervous system tumour, intracranial vascular abnormality, prior intracranial haemorrhage.
There is no Recent GI bleed or major surgery
The patient is not at risk of bradycardia
Patient does not have dialysis or severe liver disease
Patient tolerated Aspirin and Ticagrelor for 12 months with no problems.
No history of spontaneous bleeding requiring medical attention.

**The recommendation was made by**: Abigail Barrowcliff Advanced Cardiology Pharmacist, LTHT according to a protocol agreed with Dr Rani Khatib, Consultant Cardiology Pharmacist , Dr S Wheatcroft, consultant interventionist cardiologists and Prof Alistair Hall, consultant cardiologist, in line with NICE TA420 and ESC guidelines on extended DAPT 2017.
